# Laboratory-scale method for enzymatic saccharification of lignocellulosic biomass at high-solids loadings

**DOI:** 10.1186/1754-6834-2-28

**Published:** 2009-11-04

**Authors:** Christine M Roche, Clare J Dibble, Jonathan J Stickel

**Affiliations:** 1 National Renewable Energy Laboratory, National Bioenergy Center, 1617 Cole Boulevard, Golden, CO 80401-3393, USA; 2 Current address: University of California-Berkeley, Department of Chemical Engineering, 201 Gilman Hall, Berkeley, CA 94720-1462, USA

## Abstract

**Background:**

Screening new lignocellulosic biomass pretreatments and advanced enzyme systems at process relevant conditions is a key factor in the development of economically viable lignocellulosic ethanol. Shake flasks, the reaction vessel commonly used for screening enzymatic saccharifications of cellulosic biomass, do not provide adequate mixing at high-solids concentrations when shaking is not supplemented with hand mixing.

**Results:**

We identified roller bottle reactors (RBRs) as laboratory-scale reaction vessels that can provide adequate mixing for enzymatic saccharifications at high-solids biomass loadings without any additional hand mixing. Using the RBRs, we developed a method for screening both pretreated biomass and enzyme systems at process-relevant conditions. RBRs were shown to be scalable between 125 mL and 2 L. Results from enzymatic saccharifications of five biomass pretreatments of different severities and two enzyme preparations suggest that this system will work well for a variety of biomass substrates and enzyme systems. A study of intermittent mixing regimes suggests that mass transfer limitations of enzymatic saccharifications at high-solids loadings are significant but can be mitigated with a relatively low amount of mixing input.

**Conclusion:**

Effective initial mixing to promote good enzyme distribution and continued, but not necessarily continuous, mixing is necessary in order to facilitate high biomass conversion rates. The simplicity and robustness of the bench-scale RBR system, combined with its ability to accommodate numerous reaction vessels, will be useful in screening new biomass pretreatments and advanced enzyme systems at high-solids loadings.

## Background

As the demand for non-petroleum based fuels continues to grow, more emphasis will be placed on producing a cost-competitive liquid transportation biofuel such as ethanol. One clean and renewable domestic energy source that can feasibly displace a significant fraction of petroleum usage in the USA is ethanol produced from lignocellulosic biomass [1-3]. Although large-scale ethanol production is not a new concept, converting lignocellulosic biomass to ethanol is not a trivial matter. Significant challenges lie with hydrolysis of biomass into fermentable sugars. Advanced conversion technologies must be developed to allow for the efficient conversion of lignocellulosic biomass to ethanol [4,5].

Research indicates that a chemical pretreatment followed by enzymatic hydrolysis increases the overall saccharification efficiency [6,7]. A promising approach to improving process economics involves increasing biomass concentration in both pretreatment and enzymatic hydrolysis. Higher starting biomass substrate concentrations lead to higher product concentrations throughout the production process. This will result in reduced capital and production costs associated with the reduction of equipment size and energy usage for heating, cooling and mixing. While a few high-solids enzymatic hydrolysis studies have been conducted, including experiments with rotating horizontal reactor vessels, most have only been at scales ≥ 1 L [4,8-12]. To study the synergisms between pretreatment and enzymatic hydrolysis at process relevant conditions, it is necessary to be able to effectively screen both pretreated biomass and enzyme preparations at high-solids loadings (≥ 15% insoluble solids) in small-scale reactors.

Typically, shake flasks (SFs) are used for screening both pretreated biomass for enzyme digestibility and enzyme preparations for efficiency and effectiveness in digesting biomass at the National Renewable Energy Laboratory (NREL) and elsewhere. However, this is usually performed at a low insoluble solids loading (< 10%). At high insoluble solids loadings (≥ 15%), mixing modes that require bulk fluidity, such as conventional stirring or shaking, become ineffective [10,11]. Mass transfer limitations that occur with ineffective mixing, such as poor enzyme distribution and localized hydrolysis product build-up, confound saccharification screening results and must, therefore, be mitigated.

In this study, we compared small-scale enzymatic saccharification vessels with three different mixing mechanisms: shaking, gravitational tumbling and hand stirring. This comparison assessed the reaction systems for their efficiency and repeatability in converting biomass at high-solids loadings, where biomass conversion was the measure of effectiveness of enzymatic saccharification. When biomass and enzyme are effectively mixed, yield is similar, regardless of reactor system. The roller bottle reactor (RBR) system was well mixed in every instance of continuous rolling. Therefore, our results showed that a high-solids enzymatic saccharification method requiring the least user intervention (that is the RBR) would be the most efficient system for this work. Using this method, bench and floor scale enzymatic saccharifications verified the scalability of the reactor system. A few different intermittent mixing modes were also studied. In addition, we present a general equation for calculating conversion in high-solids systems and an extension of that equation to include significant, strategically chosen biomass hydrolysis products. This method, including the general conversion equation, can be applied to screen a wide variety of pretreated biomass, as well as enzyme preparations at high-solids loadings.

## Methods

### Biomass substrate

Lignocellulosic biomass in the form of corn stover was the substrate in this study. The corn stover was pretreated using dilute sulphuric acid in three separate reactors - a continuous 1 ton/day pilot-scale vertical reactor (VR), a 4-L steam explosion (SE) batch reactor and a continuous 200 kg/day horizontal reactor (HR) - for a total of five conditions. Pretreatment methods in NREL's continuous VR and SE batch reactor have been reported by Schell *et al*. [13] and Nguyen *et al*. [14], respectively. A two-stage acid hydrolysis analytical method was used to determine the carbohydrate and lignin composition of the pretreated material [15]. Table 1 summarizes the pretreatment conditions and the resulting xylan conversion. Prior to enzymatic hydrolysis, water-soluble hydrolyzate liquors were removed from the whole pretreated slurry by thoroughly washing the pretreated corn stover (PCS) with deionized water. High-solids concentrations (> 20%) were achieved by centrifugation in order to remove the majority of the excess water and, if necessary, further water was removed by pressing the solids in a custom-built hydraulic filter-press. The insoluble solids content was determined in duplicate, using HR83 Halogen moisture analysers (Metler Toledo, OH, USA). All enzymatic saccharification reactions use pretreatment sample number 1 (PCS 1) unless otherwise noted.

**Table 1:** Pretreatment conditions and yields.

Sample	1	2	3	4	5
Reactor	VR	SE	SE	HR	HR
Acid concentration*	48	7.25	7.25	4.5	2.5
pH	1.3	1.6 to 2.7†	1.6 to 2.2†	2.2	2.3
Reactor Temperature	190°C	170°C	150°C	150°C	150°C
Residence time	1 min	30 min	30 min	3 to 5 min‡	2 to 4 min‡
Combined Severity§	1.3	0.8 to 1.9	0.8 to 1.3	-0.2 to 0	-0.5 to -0.2

Xylan removed	88.9%	88.9%	86.7%	83.1%	19.6%
Monomeric xylan Yield	72.1%	69.6%	61.3%	53.4%	2.1%
Total xylan yield	81.4%	79.2%	83.4%	79.9%	19.1%
Furfural yield	9.7%	9.7%	3.3%	3.3%	0.5%

Cellulose content	59.1%	62.9%	59.1%	57.0%	42.2%

*Concentration in mg Acid/g dry biomass.

† Addition of steam throughout reaction dilutes the sample and changes the pH.

‡Residence time distribution (± 2 standard deviations).

§Calculated as reported by van Walsum [21]

### Reaction vessels and mixing modes

Three small-scale reaction systems with different mixing modes (shaking, gravitational tumbling and hand stirring) were evaluated for their efficiency and consistency in converting high-solids loadings of biomass. For mixing via shaking, experiments were performed in 250 mL wide-mouth Pyrex bottles (Thermo Fisher Scientific, Inc, MA, USA). A rotary shaking incubator (New Brunswick Scientific, NJ, USA) provided mixing at 130 rpm and maintained the temperature of the SFs. The SFs were not homogenized or hand-mixed before sampling unless specified in the mixing-mode description. For gravitational tumbling and hand stirring, experiments were performed in wide-mouth polypropylene bottles of two sizes: 125 mL and 250 mL (Thermo Fisher Scientific, Inc, MA, USA). The reaction vessels that were mixed via gravitational tumbling, denoted as RBRs, rotated horizontally at 2 rpm for 250 mL bottles and 4 rpm for 125 mL bottles on a three deck roller apparatus for mini bottles (Wheaton Industries Inc, NJ, USA). Previous work showed that mixing speed does not affect the biomass conversion in the range of 2-20 rpm [12]. Temperature control was achieved by housing the roller apparatus in a general purpose incubator (Model 1545, VWR International, LLC, PA, USA). The hand-mixed reaction vessels (HMR) were stirred with a sterilized spatula for 30 s at each prescribed mixing time and were incubated standing vertical in a stationary incubator.

### Enzymatic hydrolysis

Inside a laminar flow hood, autoclaved or ethanol sterilized SFs, RBRs and HMRs were loaded with a gram mass that was equal to half of the volume capacity of the vessel in millilitres (for example, 125 g into a 250 mL vessel). Of that mass, reactors were charged with 15%, 20% or 30% insoluble solids and 50 mM (pH 4.8) sterile filtered citrate buffer. Tetracycline (10 μg/mL) was added to inhibit microbial contamination. Two enzyme preparations were used for our studies: spezyme CP [Lot No. 301-05021-011] and GC220 [Lot No. 4900759448] (Genencor-Danisco, NY, USA). For spezyme CP and GC220, respectively, the total protein was assayed at 134 mg/mL and 202 mg/mL (BCA assay, Pierce Biotechnology, Inc, IL, USA) and the specific activities were determined to be 0.49 FPU/mg protein and 0.60 FPU/mg protein using the NREL laboratory analytical procedure "Measurement of cellulase activities" [16]. The enzyme preparations were sterile filtered prior to loading into the reaction vessels. The enzyme was loaded at 5, 10, 15, 20, or 30 mg protein/g cellulose. The cellulose content of each pretreated material is summarized in Table 1.

Reactors loaded with PCS, the buffer and the antimicrobial agent were brought to 48°C, while mixing, before adding the enzyme. The enzyme was distributed on the surface of the PCS slurry across the length of the vessel. The enzyme was not mechanically mixed into the RBR or the SF unless specified. For 250 mL vessels, enzymatic saccharification slurry samples (~1 mL for 15% and 20% initial insoluble solids loadings and ~2 mL for 30% insoluble solids loadings) were taken every 4 hours for the first 8 hours and then once a day thereafter for a total of 7 days. Saccharifications reactions performed in 125 mL vessels were sampled at 8 hours, 1 day, 2 days, 4 days and 7 days. Reactor sampling was performed under sterile conditions in a laminar flow hood using standard aseptic techniques. The samples were not homogenized by hand mixing prior to sampling for the RBRs or for the SFs unless specified. Although our avoidance of hand mixing is unconventional, it was necessary in order to prevent mixing in addition to the primary mixing mechanism of study.

### Compositional analysis

Enzymatic hydrolysis slurry samples were centrifuged in 0.45 μm nylon membrane microcentrifuge filters (No. ODM45C35, Pall Corp, MI, USA) at 12,500 rpm for 5 min. The filtered liquid was diluted 1:5 with deionized water in high-performance liquid chromatography (HPLC) vials for subsequent analysis. Samples were run on an Agilent 1100 series HPLC with a Shodex SP0810 Sugar Column (Kawasaki, Japan) run at 85°C. Deionized water pumped at 0.6 mL/min was the eluent.

The density of the end-point hydrolyzate slurry liquid fraction (*ρ*_*l*_) was measured on a density meter (DMA5000, Anton Paar, VA, USA). Values for mass fraction insoluble solids (*f*_*is*_) of the slurry at the end of the reaction were measured using a direct calculation method [17]. Briefly, the mass fraction total solids (soluble and insoluble) of the hydrolysis slurry and the mass fraction soluble solids in the separated liquid were determined using HR83 halogen moisture analysers, and the *f*_*is *_of the hydrolysis slurry was calculated from these measures with mass balance relationships.

### Yield calculations

Typically, yield or percent conversion is used to quantify the effectiveness of enzymatic hydrolysis of biomass. In general, this can be calculated by dividing the amount of hydrolyzed biomass by the amount of initial hydrolyzable biomass. The following general equation can be used to calculate conversion for both simple and complex systems:(1)

where *r*_*j*, *i *_is the molecular weight (MW) ratio of the polysaccharide *j *to its respective mono-or oligo-saccharide *i *(for example, *r*_*G*, *cb *_is two glucan units to cellobiose [324.32/342.34]), *f*_*i *_is the mass fraction of component *i *as a part of the total slurry [*g i/g *slurry], *x*_*j *_is the mass fraction of *j *in the insoluble solids [*g j/g *insoluble solids], *N *is the number of hydrolyzed components considered, *M *is the number of insoluble solids hydrolyzable components considered, Δ denotes a change from the initial conditions and the subscripts *is *and 0 refer to insoluble solids and initial condition, respectively.

As discussed in prior work [12], biomass conversion calculations can be formulated to include as few or as many components as desired. A simple cellulose conversion equation may only consider glucose and, possibly, cellobiose as a conversion product [9]. A complex cellulose conversion calculation can be expanded to include larger glucose oligomers as products by adding the respective mass fractions to the numerator. Additionally, a hemicellulose conversion can be considered along with a cellulose conversion to quantify the effectiveness of biomass enzymatic hydrolysis. Together, the following conversion equation for cellulose and hemicellulose can be described from major breakdown products:(2)

where the subscripts refer to the following: *C *is for cellulose, *H *is for hemicellulose, *G *is for glucan, *g *is for glucose, *cb *is for cellobiose, *X *is for xylan, *x *is for xylose, *L *is for galactan, *l *is for galactose, *A *is for arabinan and *a *is for arabinose. For the enzymatic hydrolysis system studied in this work, we will look at cellulose conversion based on glucose and cellobiose yields as given by:(3)

As discussed in previous work, conversion equations for high-solids biomass saccharification systems require the sugar concentrations to be on the basis of a mass fraction of the whole slurry in order to prevent an over-estimation of the yield [9,12,18]. Liquid concentrations (*c*) in [*g/L*] can be related to mass fraction (*f*) in [*g/g *slurry] by:(4)

where *ρ*_*l *_is the density of liquid. Hodge *et al*. [9] derived an equation that allows the estimation of *f*_*is *_from liquid sugar concentrations and is given as:(5)

The following equation estimates *ρ*_*l *_using linear interpolation(6)

where *m *is the density additive amount for concentrations of glucose, cellobiose and xylose, which was determined as 0.456, and *ρ*_*l*,0 _is the initial density of the liquid fraction of the saccharification slurry. Xylose is included in the *f*_*is *_and *ρ*_*l *_estimations because the xylan conversion to xylose contributes significantly to the decrease in *f*_*is *_and increase in liquid density [12].

### Uncertainty analysis

An uncertainty analysis of random errors was performed for all the enzymatic saccharification data sets. Two to four saccharification experiments were performed per condition. At each sampling time-point per condition, the term-of-interest average value was determined. The standard deviation of the difference of the measured or calculated values from the average was found for all time-points, collectively, within a condition. The change of standard deviation over reaction progression was determined to be negligible and, therefore, we assumed constant uncertainty for an experimental condition over the entire enzymatic saccharification reaction. Uncertainty of the averaged values was calculated using the following:(7)

where *t *= *t*_(*ν*, 95) _is the 95% *t*-value corresponding to *ν *= *N *- 1 degrees of freedom, *S*_*x *_is the standard deviation, and *N *is the number of values. The uncertainty values calculated in this way were used for the error bars in the figures.

## Results and discussion

### Evaluation of reactor type

**Figure 1:**
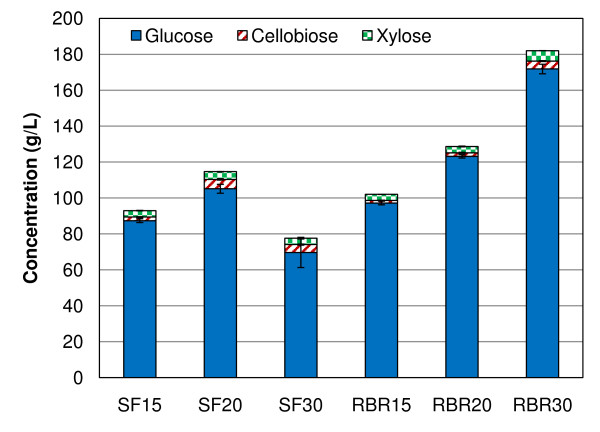
**Sugar concentrations**. Glucose, cellobiose, and xylose liquid phase concentrations after 7 days of enzymatic saccharification in shake flasks and roller bottle reactors at 15%, 20% and 30% initial insoluble solids.

**Figure 2:**
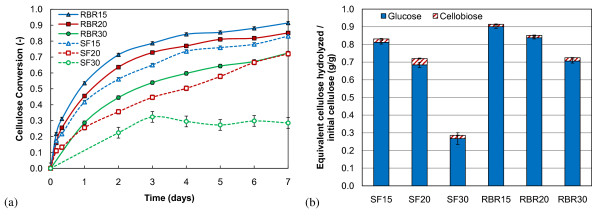
**Shake flasks (SFs) and roller bottle reactors (RBRs)**. (a) Cellulose conversion throughout enzymatic saccharification of pretreated corn stover at 15%, 20% and 30% initial insoluble solids and 20 mg protein/g cellulose GC220 enzyme loading in SFs and RBRs. (b) Cellulose conversion to glucose and cellobiose after 7 days of enzymatic saccharification.

In order to compare the efficiency and reliability of SFs and RBRs in enzymatic hydrolysis of high-solids biomass loadings, we performed a series of saccharification reactions in 250 mL reactors. For each reactor type, three solids loadings (15%, 20% and 30% insoluble solids) of PCS 1 were used in quadruplicate, for a total of 12 reactions. The GC220 enzyme loading was held constant at 20 mg enzyme/g cellulose for these experiments. Figure 1 shows the average liquid phase sugar concentrations from the unstirred SFs and RBRs after 7 days of hydrolysis. We observed glucose concentrations higher than 170 g/L in RBRs loaded with 30% insoluble solids. The cellulose conversion, based on cellobiose and glucose yields and calculated using Equation 3, was averaged for quadruplicate reactors at each sampling time-point and plotted with uncertainty bars at a 95% confidence interval in Figure 2(a). Figure 2(b) shows a breakdown of the sugars contributing to the cellulose conversion.

The results show that, with mixing using only the shaking action of the rotating platform, SFs give lower conversions for every insoluble solids loading tested with respect to the corresponding insoluble solids loading in RBRs. Results similar to the RBRs have been observed in SFs when the enzyme is well mixed by hand initially and before each sample, even up to 30% insoluble solids (see section **Intermittent Mixing **below). At 30% initial insoluble solids loadings, RBRs converted approximately 2.5 times as much of the potential sugar as the SFs. Not only do the SFs give lower yields but they also show greater variability, particularly with the higher solids loadings. The greater variability can probably be attributed to the lack of consistency of the shaking only mechanism among high-solids SFs, coupled with sampling of the heterogeneous mixture within each individual SF. RBRs enable a higher conversion of biomass at all insoluble solids loadings tested here than the SFs using the shaking mechanism alone. This is likely due to a more effective mixing by horizontal tumbling and, therefore, fewer mass transfer limitations and localized product build-up. By visual observation, the 30% initial insoluble solids RBRs began to liquefy within 4 hours, whereas it took the unstirred SFs nearly 6 days to begin to liquefy.

### Reactor scale

**Figure 3:**
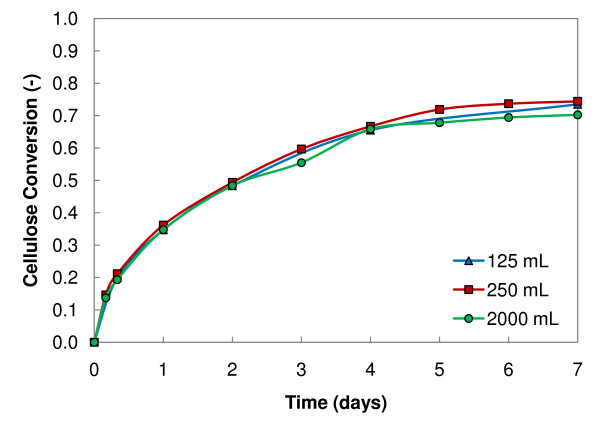
**Reactor scale**. Cellulose conversion throughout enzymatic saccharification in 125 mL, 250 mL, and 2 L roller bottle reactors (RBRs) loaded with 20% initial insoluble solids and 20 mg protein/g cellulose spezyme CP. The data for the 2 L RBRs are from [12].

In developing a high-solids enzymatic saccharification methodology, the RBR was identified as a reaction vessel that provides effective mixing at a small scale. Depending on the energy cost for mixing by tumbling, either by the rotation of the entire vessel or by internal rotating paddles, a horizontal reaction vessel may be of potential use at pilot and manufacturing scales. A second set of experiments determined the effect of the RBR scale on their efficiency in enzymatic saccharification of high-solids biomass loadings. Saccharification reactions were performed in quadruplicate in 125 mL and 250 mL RBRs with all chemical conditions held constant (20% initial insoluble solids loading and 20 mg protein/g cellulose loading of spezyme CP enzyme). Cellulose conversion values from these experiments were averaged at each sampling time-point and plotted in Figure 3 with data obtained in prior experiments of 2 L RBRs run under the same experimental conditions, as previously reported [12]. Uncertainty error bars of 95% confidence intervals are included but are not larger than the data point size. As shown in Figure 3, the enzymatic saccharification reactions do not appreciably differ and, thus, for conditions tested, RBRs are scalable between 125 mL and 2 L. Roche and co-workers [12] also showed that equivalent results were obtained for rotating the entire vessel or for rotating internal paddles. These results show promise for further scale-up of this reaction vessel, provided the economics of a full-scale vessel are favourable.

### Biomass pretreatment and enzyme preparation screening

**Figure 4:**
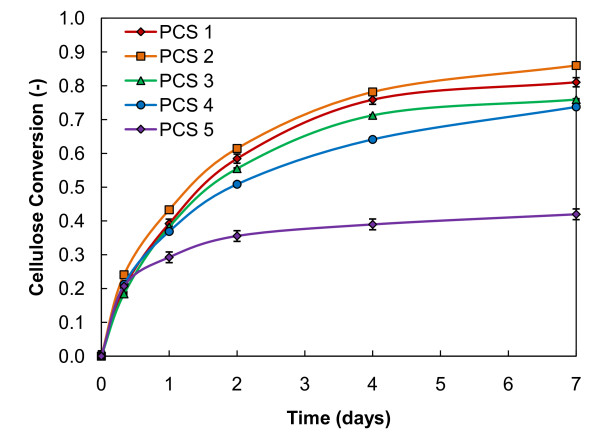
**Biomass pretreatments**. Cellulose conversion throughout enzymatic saccharification for different pretreated corn stove (PCS) samples in continuously mixed roller bottle reactors loaded with 20% initial insoluble solids and 20 mg protein/g cellulose GC220. PCS 5 was loaded with 30 mg protein/g cellulose instead of 20 mg/g. Refer to Table 1 for pretreatment conditions used.

To evaluate our high-solids enzymatic saccharification method, we tested five samples of dilute-acid pretreated corn stover. RBRs were loaded in duplicate with 20% initial insoluble solids and 20 mg protein/g cellulose of GC220 enzyme, except for PCS 5 which was loaded with 30 mg protein/g cellulose of GC220 enzyme. Cellulose conversion was averaged for each sample time-point and plotted with 95% uncertainty bars in Figure 4. The results show clear differences in the digestibility among the five different pretreatment conditions, with PCS 2 reaching 86% cellulose conversion. Even with a higher enzyme loading, the cellulose conversion of PCS 5 is nearly 50% lower than all other PCS tested. This is possibly related to the mild pretreatment conditions that solubilized very little xylan (Table 1). With less xylan removal, cellulose is less available for enzymatic attack [19,20]. Although the enzymatic conversion results shown in Figure 4 do not strictly relate to the amount of xylan removed during pretreatment, in general this relationship is observed. All severity levels were observed to tumble in the RBRs and efficient mixing was promoted. The more fibrous nature and larger particle size (mean diameter of 773 μm) of the lower-severity PCS did not appear to adversely affect the mixing ability of the roller bottle system.

**Figure 5:**
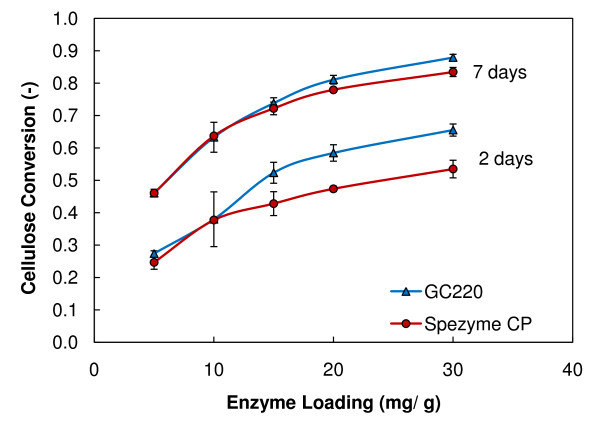
**Enzyme preparations**. Enzyme loading curves of GC220 and spezyme CP at 2 and 7 days of saccharification in roller bottle reactors loaded with 20% initial insoluble solids.

In addition to testing several pretreated biomass samples, we studied two enzyme preparations. Again, PCS 1 was loaded into duplicate RBRs at 20% initial insoluble solids with 5, 10, 15, 20, or 30 mg protein/g cellulose loading of GC220 or spezyme CP enzyme. In Figure 5, the enzyme loading curves are plotted with average cellulose conversion values after 2 and 7 days of enzymatic saccharification. After 7 days of enzymatic saccharification, the two enzyme preparations show little difference up to a 20 mg protein/g cellulose enzyme loading. At 2 days, enzyme loadings greater than 10 mg protein/g cellulose exhibit a greater difference of saccharification. The results indicate that GC220 has a higher initial rate of cellulose conversion, but the better performance of GC220 compared to spezyme CP is less pronounced after longer saccharification times.

### Intermittent mixing

In order to examine minimum mixing requirements for adequate mass transfer, we looked at several intermittent mixing modes: hand mix at time zero (HMR t0); hand mix at time zero; and then hand mix once per day (HMR t0, 1pd), shake flask with hand mix at time zero and then at each sampling (SF; HMR t0, sample), roll for 4 hours after time zero and then roll for 1 hour per day (RBR 4 hr, 1hrpd), roll for 4 hours after time zero and then roll for 1 hour per 2 days (RBR 4 hr, 1hrp2d), and roll for 24 hours after time zero and then shake for remainder (RBR 24 hr, SF). This set of experiments was run in quadruplicate in 125 mL HMRs and RBRs. For these experiments, all chemical conditions were held constant at 20% initial insoluble solids of PCS 1 and 20 mg protein/g cellulose GC220 enzyme loading. The hand-mixed SF experiments (SF; HMR t0, sample) were run separately in 250 ml wide-mouth Pyrex bottles filled with 100 g of biomass.

**Figure 6:**
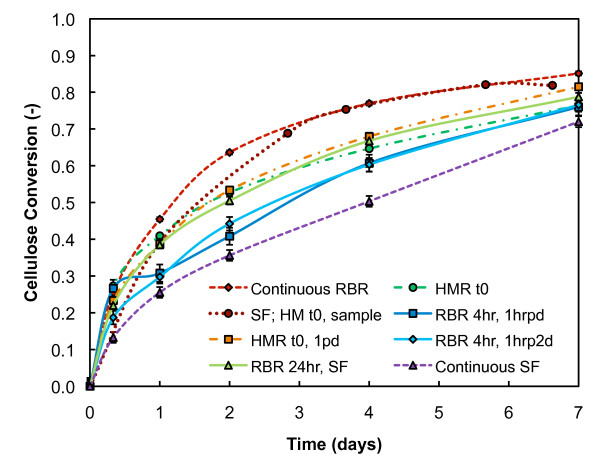
**Intermittent mixing**. Cellulose conversion throughout enzymatic saccharification of intermittently mixed reaction vessels loaded with 20% initial insoluble solids and 20 mg protein/g cellulose GC220. See the text for descriptions of each mixing mode.

Cellulose conversion values, with uncertainty bars of a 95% confidence interval, are plotted in Figure 6. The average cellulose conversion profile of both continuously rotated RBRs and continuously shaken, not stirred, SFs, which were run at the same chemical conditions as the intermittently mixed saccharification vessels, are also included in Figure 6 for comparison. As expected, the cellulose conversions of the intermittently mixed saccharifications lie within the high and low bounds of the mixing effectiveness extremes; that is, the effective mixing of the continuous RBR and the ineffective mixing of the continuous SF with no hand mixing. For RBRs that initially rolled for 4 hours and then intermittently afterward (1 hour per 1 or 2 days), some variability is observed in the conversion profile, especially at early reaction times. The inconsistencies can be attributed to sampling variability. These RBR experiments with limited initial rolling did not receive effective initial distribution of the enzyme and, therefore, were heterogeneous in nature, with localized high and low product concentration. All of the other conditions appeared to be more homogeneous, especially at early time points, which was most likely due to more effective mixing and better initial enzyme distribution.

The data indicate conversion trends for the specific mixing modes. The rate of enzymatic hydrolysis is higher for the conditions that are mixed well early in the experiment, experiencing better conversions than conditions with less initial mixing. For conditions that received at least intermittent mixing throughout the 7 days of enzymatic saccharification, we observed a higher continued rate of conversion compared to saccharifications with no further mixing beyond the initial mixing. The experiment that was hand-stirred only initially exhibits the first conversion trend of a high initial rate of conversion, while the RBRs that were mixed 4 hourly initially, and intermittently thereafter, follow the second conversion trend of continued higher conversion later in the reaction. The intermittent mixing conditions, HMR that was mixed at time zero and once per day thereafter and RBR that rolled for 24 hours and shaken for the remainder, exhibited both of these behaviours. The hand-mixed SF experiment was not sampled at 8 hours and, therefore, it is not possible to determine the initial rate.

The hand-mixed SF mixing mode provided statistically equivalent cellulose conversions as the continuous roller-bottle mixing mode, both for 20% initial insoluble solids (Figure 6) and 30% initial insoluble solids (data not shown). Although not always documented in experimental procedures, it is customary to hand-mix the biomass after enzyme addition to and before sampling from SFs when experiments with high-solids loadings are performed. It is important to note that, in the hand-mixed SF method, hand mixing is a more prominent mixing mode than shaking for the high-solids conditions early in the saccharification reaction. However, hand mixing is a somewhat ill-defined mixing mode, in that its effectiveness will depend on how frequently the hand mixing is performed, and hand mixing would be difficult to replicate at pilot and manufacturing scales. Conversely, the continuous roller-bottle mixing mode is well defined and there is precedent for mixing by rotation at larger scales (for example, cement mixers).

## Conclusion

Horizontally rotating bottles for the enzymatic saccharification of biomass has proved to be a more significantly consistent reactor system than SFs at high-solids loadings. The gravitational tumbling achieved in the RBRs provided sufficient mixing throughout the entire reaction vessel, thus mitigating mass transfer limitations that may confound enzymatic saccharification results. Using small-scale, continuously rotating bottle reactors, we have developed a method for screening pretreated biomass for enzyme digestibility and enzyme preparations for effectiveness in digesting biomass at process relevant, high-solids conditions. The reactor system and method was shown to be scalable between bench (125 mL) and floor (2 L) scales at 20% initial insoluble solids loadings. As a part of this enzymatic saccharification method, we generalized and expanded previously developed yield calculations that account for the multiphase nature of the high-solids enzymatic saccharification slurry. The results support our hypothesis that this reactor system will work well for a variety of biomass types and pretreatment severities at high-solids conditions. The simplicity of this reactor system and its ability to accommodate numerous reaction vessels will be useful in screening new biomass pretreatments and advanced enzyme systems at high-solids loadings.

Intermittent mixing regimes for the RBR and hand-stirred systems were explored to determine whether adequate conversion can still be obtained with substantially less total mixing, thus reducing energy costs. Effective initial mixing to promote good enzyme distribution and continued, but not necessarily continuous, mixing is required to facilitate high conversion rates.

## Abbreviations

HMR: hand-mixed reaction vessel; HPLC: high-performance liquid chromatography; HR: horizontal reactor; MW: molecular weight; NREL: National Renewable Energy Laboratory; PCS: pretreated corn stover; RBR: roller bottle reactor; SE: steam explosion; SF: shake flask; VR: vertical reactor.

## Competing interests

The authors declare that they have no competing interests.

## Authors' contributions

CMR designed, coordinated and performed the enzymatic saccharification experiments, analysed the results and drafted the manuscript. CJD participated in the design of the study and performed enzymatic saccharifications. JJS contributed to the original conception of the study and advised on the design and progress of the experimentation. All authors critically revised the draft and approved the final manuscript.
